# Progress in Radiotherapy

**Published:** 1903-02-14

**Authors:** 


### PROGRESS IN RADIOTHERAPY.

In a paper read before the Dermatological Section of the British Medical Association, Freund,[1] of Vienna, discusses radiotherapy and phototherapy He considers all radiant phenomena have the same physical basis—that the effect of the rays upon the body varies with the dosage, from mere stimulation favouring organic processes to an actual destruction of tissues, the vitality being lowered. Radiant heat, light, electricity, and *x*-rays similarly influence cell-life. Clinically the physiological effects are in direct proportion to the intensity of the raying, but inversely proportional to the wave-length. The latent period of the reaction obtained is inversely proportional to wave-length and dose. The most powerful in producing fluorescence are the most powerful physiologically. The use of high-frequency currents falls under the head of radiotherapy : the physiological effects are due, in his opinion, to the spark discharges ; these producing the effects by (*a*) mechanical bombardment of the tissues, (*b*) the production of heat, (*c*) chemical effects—formation of ozone, (*d*) ultra-violet ray formation.

The high-frequency currents are useful in various forms of pruritus and in lupus erythematosus.

The *x*-rays are useful, by removing the hair, in ringworm, favus, sycosis, hypertrichosis. The *x*-rays have no bactericidal properties, but act by direct destruction of tissue or alteration of the blood supply of the follicles. In lupus and epithelioma they act by destruction, by promotion of cicatrices and the formation of connective tissue, or directly upon the specific toxin. In lupus vulgaris the results of *x*-ray and Finsen methods give equally good cosmetic appearances. A rational method would be to *x*-ray large surfaces and use the Finsen method for remaining foci. With "soft" tubes the reaction is longer delayed than with hard ; the latter then are probably safer.

The clinical effects of *x*-raying are (1) intumescence of skin, (2) mild erythema, (3) pigment changes, (4) loosening of hairs, (5) subjective phenomena—itching, burning, etc.

The therapeutic value of Finsen's apparatus depends not only upon the rays of shorter wave-length, but also on those of longer length and more penetrating power.

Sequeira[2] considers that in rodent ulcer wide excision is the best treatment where possible, but where this cannot be carried out the *x*-rays are of the greatest service. Of 80 cases under treatment 34 were cured, and most of the remainder were still under treatment. Recurrent nodules were common, but could be dispersed by the same treatment. Where cartilage, periosteum, or bone were affected the results were not good. A low tube can be used for removing stubborn hard edges.

Lupus may be cured by the *x*-rays, but relapses occur again and again. In lupus erythematosus the *x*-rays are valueless.

Dore[3] states that cases can be treated by the Finsen light indefinitely without disfigurement. The reactions can be increased greatly by the use of pyrogallol or acid nitrate of mercury.

McLeod[4] finds that a 1 per cent. solution of permanganate of potash, or a weak solution of iodine with glacial acetic acid, or pure carbolic acid, greatly increase this reaction. Norman Walker[5] has also found pure carbolic acid useful as an adjunct to the Finsen treatment. Abraham[6] considers that *x*-rays are especially useful for mucous membranes. He has cured a case of lupus erythematosus by this method, though in some cases the disease is made distinctly worse.

Professor Wild's[7] experience with light and lupus erythematosus were not encouraging, but Norman Walker[8] finds improvement follow in many cases with the *x*-rays. The latter and Dr. Brooke[9] have found good results follow the use of the *x*-rays in cases of mycosis fungoides.

Brooke suggests scarifying refractory nodules of rodent ulcer superficially. This helps in making them disappear. While, after injections of tuberculin for lupus, Hall-Edwards[10] has seen glands enlarged and indurated, he has not seen the glands affected subsequent upon the light or *x*-ray treatment in any case which has come under his notice.

In his hands the *x*-rays have given the best results even for lupus. In this method of treatment he is of the opinion that a hard tube is best, and holds that electrostatic or electrolytic action plays some part in the production of the results. He finds that with the use of metal masks the metal becomes charged with electricity, which occasionally will discharge on to the patient's skin where in contact, and lead to a dermatitis. He suggests, to remedy this, that the tube be placed in a box lined with an opaque substance, *e.g.* indiarubber, leaving a round aperture in front to which can be fitted a cardboard or vulcanite tube lined with an opaque application, such as bismuth in gelatine, to within an inch of the free ends. The advantages of this method are that the patient can hold the affected part to the rays without the danger of getting too close. Suitable diaphragms will lessen the aperture for smaller areas.

He differs from Albers Schönberg, who lays great stress on the undesirability of setting up a dermatitis. He holds that such ulceration is superficial and quickly heals. He has had good results with the mucous membranes of the lips, but finds with Schönberg no good ensue on the nasal mucous membrane. He speaks hopefully of the treatment of cancer. Pain, untouched by drugs, has disappeared under treatment, even though the disease is too far advanced for successful treatment. Dr. Morton,[11] of New York, has published the same result.

The use of the brush discharge coincidently with the $x$-rays is recommended by Snow[12] for treatment purposes.

[1] Brit. Med. Jour., Oct. 25, or Lancet, Aug. 2. [2] Brit. Med. Jour., Oct. 25. [3] Ibid. [4] Ibid. [5] Ibid. [6] Ibid. [7] Ibid. [8] Ibid. [9] Ibid. [10] Bir. Med. Review, June. [11] Med. Rec., May 24. [12] Ibid., Sept. 20.

*(To be continued.)*

